# Association Between Introduction of the 23-valent Pneumococcal Polysaccharide Vaccine (PPSV23) and Pneumonia Incidence and Mortality Among General Older Population in Japan: A Community-based Study

**DOI:** 10.2188/jea.JE20240285

**Published:** 2025-05-05

**Authors:** Aya Sugiyama, Masaaki Kataoka, Kentaro Tokumo, Kanon Abe, Hirohito Imada, Bunlorn Sun, Golda Ataa Akuffo, Tomoyuki Akita, Shingo Fukuma, Noboru Hattori, Junko Tanaka

**Affiliations:** 1 Department of Epidemiology, Infectious Disease Control and Prevention, Graduate School of Biomedical and Health Sciences, Hiroshima University, Hiroshima, Japan; 2 Project Research Center For Epidemiological & Mega-Data Analysis of New Research Area, Hiroshima University, Hiroshima, Japan; 3 Sera Central Public Hospital, Hiroshima, Japan; 4 Department of Molecular and Internal Medicine, Graduate School of Biomedical and Health Sciences, Hiroshima University, Hiroshima, Japan; 5 Hiroshima University Hospital, Department of Clinical Oncology, Hiroshima, Japan

**Keywords:** pneumococcal vaccination, elderly, incidence, mortality, interrupted time series analysis

## Abstract

**Background:**

With global aging, especially in Asia, preventing pneumonia among seniors is vital. The necessity of introducing pneumococcal vaccines among the elderly has been highlighted, but there is a paucity of community-based real-world evidence on their effect. Sera Town in Hiroshima Prefecture, a super-aged community, launched a distinctive pneumococcal vaccination support project for elderly residents and conducted a 5-year follow-up survey. This study evaluates the effectiveness of this vaccination initiative.

**Methods:**

From October 2010 to March 2015, Sera Town recruited elderly residents for PPSV23 vaccination with partial cost subsidies. Participants were surveyed annually for 5 years post-vaccination to assess pneumonia incidence, calculated on a person-years basis. Using vital statistics from 2000 to 2016, we quantified changes in mortality rates associated with the vaccination support project through an interrupted-time-series analysis.

**Results:**

Of approximately 7,900 residents aged 65 and older, 3,422 (43%) participated in the project (median age: 84 years; range: 70–114 years; 56.7% female). Over 14,559 person-years of observation, 295 participants developed pneumonia. The post-vaccination incidence rate was 20.3 per 1,000 person-years (95% confidence interval [CI], 18.0–22.7). Interrupted time series analysis indicated a 25% reduction in Sera Town’s pneumonia mortality rate post-project, reversing an annual increase of 0.23 per 1,000 population pre-project to an annual decrease of 0.04 per 1,000 population post-project.

**Conclusion:**

This study provided real-world evidence on the association with PPSV23 vaccination on the general elderly through a community-based study. The results may be particularly useful for regions where PPSV23 serotypes are prevalent, offering insights for areas facing aging challenges.

## INTRODUCTION

Pneumonia is one of the leading causes of death in Japan, ranking fifth after malignant neoplasms, heart diseases, senility, and cerebrovascular diseases (60.6 per 100,000 population).^1^ The number of deaths from pneumonia in Japan reaches approximately 74,000 annually, with 98% of these deaths occurring in individuals aged 65 and older.^1^ The primary causative bacterium of pneumonia is Streptococcus pneumoniae. Vaccination against pneumococcal pneumonia in the elderly is expected to significantly contribute to the prevention of pneumonia and reduction in mortality rates. The World Health Organization (WHO) recommends prioritizing the introduction and enhancement of pneumococcal vaccination programs for children.^2^ In countries with well-established pediatric vaccination programs, the WHO advises considering the implementation of vaccination programs for the elderly. In Japan, the 13-valent pneumococcal conjugate vaccine (PCV13) was introduced into the National Immunization Program for children in 2013, achieving a coverage rate of 97–99%.^3^ For the elderly, a nationwide routine vaccination program under the National Immunization Program with the 23-valent pneumococcal polysaccharide vaccine (PPSV23) began in October 2014. However, the vaccination coverage rate is reported to be between 19.8% and 21.7%.^4^

**Figure 1.**
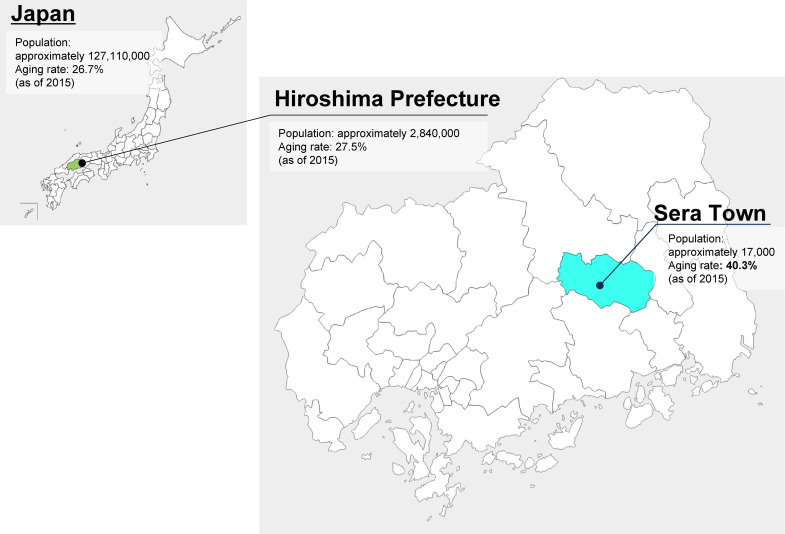
Map of Hiroshima Prefecture and Sera Town

Sera Town, located in the mountainous region of eastern Hiroshima Prefecture, is a super-aged town with a population of approximately 17,000 and an aging rate of 40.3% as of 2015 (Figure 1).^1^ This town pioneered a unique pneumococcal vaccination support project for all residents aged 65 years and older, starting in October 2010. The vaccine used was PPSV23, which was approved for use in elderly individuals in Japan at that time. The project continued until March 2015, and a 5-year follow-up survey was conducted to evaluate the association of the project in preventing pneumonia.

We analyzed data to determine the incidence of pneumonia following pneumococcal vaccination among the elderly population aged 65 years and older. Utilizing aggregated demographic data from vital statistics open datasets, we employed interrupted time series analysis (ITSA) to quantify the level and trend changes in mortality rates before and after the introduction of the pneumococcal vaccination support project in the town. The objective of this research is to provide real-world evidence on the effectiveness of pneumococcal vaccination support initiatives in a super-aged society.

## METHODS

In Sera Town, located in eastern Hiroshima Prefecture, all residents aged 65 years and older were invited to receive the pneumococcal vaccination from October 2010 to the end of March 2015. The target population during the project period was approximately 7,900 individuals. Sera Town municipality subsidized 6,000 yen of the vaccination cost, which typically amounted to around 8,000 yen. Promotion efforts included monthly features in the town newsletter distributed to all households, weekly broadcasts of a promotional video on cable TV, and incentives for local practitioners administering the vaccine. All costs were borne by the town. Vaccinated individuals were tracked for pneumonia incidence through a self-administered questionnaire survey conducted by mail once a year for 5 years after vaccination. This project was carried out with the cooperation of the Sera Town municipality, Sera Medical Association, Sera Central Public Hospital, and Hiroshima University.

### Incidence of pneumonia in individuals who participated in the pneumococcal vaccination support project

At the time of pneumococcal vaccination, the following information was collected for each participant: sex, age, body mass index (BMI), smoking history, respiratory diseases, and performance status (PS). Subsequently, a 5-year follow-up survey was conducted to track the incidence of pneumonia, and the results were compiled into a database. Pneumonia was assessed based on self-reported cases of having had pneumonia, without verification from medical records. The pneumonia incidence rate was calculated using the person-year method, with time starting from the point of vaccination. Incidence rates were also compared across various categories, including sex, age group, BMI, smoking history, presence of respiratory diseases, and PS.

### Changes in all-cause and pneumonia-specific mortality rates in Sera Town with those in Hiroshima Prefecture and across Japan

We extracted annual population data and crude mortality rates for all causes and pneumonia-specific causes from the Vital Statistics Annual Reports for Japan, Hiroshima Prefecture, and Sera Town from 2000 to 2016. The data extraction period ended in 2016 to ensure comparability under consistent conditions, because the definition of ‘pneumonia’ in cause-of-death statistics was revised in 2017 due to partial amendments to the ICD-10.

Using the mortality rate distribution from the 10 years prior to the introduction of the pneumococcal vaccination support project in Sera Town (2000–2009), we predicted the mortality rates that would have occurred without the project using general linear model. These predictions were then compared to the actual observed mortality rates from 2010 onwards. Similarly, for Japan and Hiroshima Prefecture, we used the mortality rate distributions from 2000 to 2009 to forecast the subsequent mortality rates and compared these predictions to the observed rates from 2010 to 2016.

### Association of the pneumococcal vaccination support project with pneumonia-specific mortality in Sera Town using interrupted time series analysis

We attempted to quantitatively demonstrate the level change and trend change in pneumonia-specific mortality before and after the initiation of the pneumococcal vaccination support project using population dynamics open data (aggregated data) to assess the overall effect of pneumonia mortality suppression on the entire population of Sera Town. ITSA, a method to evaluate the effect of interventions by examining changes in temporal trends before and after the intervention, was employed.^5^^–^^8^ Sera Town introduced the pneumococcal vaccine in 2010, ahead of the rest of the country, while Hiroshima Prefecture and the rest of Japan introduced the vaccine in 2014. Due to partial amendments to the International Classification of Diseases, 10^th^ revision (ICD-10), the available data extends only up to 2016. Consequently, for Hiroshima Prefecture and nationwide, there were only 2 years of post-intervention data available, making it impossible to perform an ITSA.

### Statistical analysis

Descriptive statistics, including mean and standard deviation (SD), frequency, and percentage (%), were computed. In calculating the incidence rate, the person-year method was used. The 95% confidence interval (CI) for the incidence rate was determined using the Poisson distribution. For comparing incidence rates between groups, a Poisson distribution-based test was employed.

In the model predicting the mortality rate if the pneumococcal vaccination support project were not initiated, a linear regression model was applied, using data Y (2000–2009), representing the years prior to the project introduction, as the explanatory variable, and the annual crude mortality rate as the dependent variable.

In the ITSA, year data Y (2000–2016), the intervention dummy variable Z (before the project started: Z = 0, after the project started: Z = 1), and the interaction term between Y and Z (Y × Z) were set as explanatory variables and the annual crude mortality rate as the dependent variable. Multiple regression analysis using the least squares method was conducted. The trend of pneumonia-specific mortality before the pneumococcal vaccination support project was calculated from the estimated value of Y, while the trend of pneumonia mortality after the project was derived from the sum of the estimated values of Y and Y × Z. The level change due to the project initiation was determined from the difference in the estimated values at the point of project initiation. Two-sided *P*-values <0.05 were considered statistically significant. Data analysis was performed using JMP Pro 16.0 software (SAS Institute Inc., Cary, NC, USA).

### Ethics approval and consent to participate

The ethics committee for epidemiological research of Hiroshima University approved this study (approval number, Eki-318). Informed consent was obtained from all participants. All study activities were performed in accordance with the Declaration of Helsinki and relevant guidelines and regulations in Japan.

## RESULTS

### Characteristics of the participants

**Figure 2.**
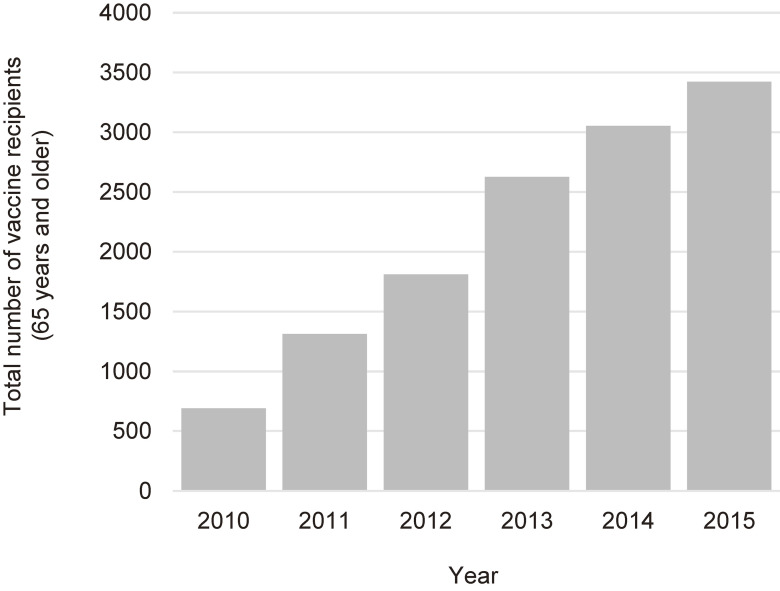
Total number of pneumococcal vaccine recipients (65 years and older) during the project in Sera Town in 2010–2015

During the implementation period of the pneumococcal vaccination support project in Sera Town, approximately 7,900 residents aged 65 years or older were targeted. Of these, 3,422 individuals participated in the project and received the pneumococcal vaccine, resulting in a participation rate of approximately 43%. The annual number of enrolled participants was shown in Figure 2. The mean age of the enrolled participants was 84.0 (SD, 8.4) years, with a median age of 84 years (range: 70–114 years). The average age at vaccination was highest in 2010 at 89.5 (SD, 6.6) years, with a trend of younger ages in subsequent years (eTable 1), suggesting that vaccination was initially prioritized for the very elderly at higher risk of pneumonia. Among the participants, 56.7% were female, 6.4% were current smokers, 24.8% were past smokers, and 18.3% had a family member who smoked (Table 1). The prevalence of respiratory diseases was 3.6% for asthma and 2.1% for chronic obstructive pulmonary disease, while the prevalence of cardiovascular diseases was 11.7%. Other comorbidities included hypertension in 45.6%, hyperlipidemia in 16.1%, diabetes mellitus in 13.1%, and stroke in 4.3%. Of the participants, 1.1% were in a nursing home, and the rest were living at home. Regarding PS, those who were able to perform normal activities (PS = 0) were the most common (84.3%).

**Table 1.** Characteristics of 3,422 residents aged 65 years and older in Sera Town who received the pneumococcal vaccine

		*N*	%
Total		3,422	100%
	
Mean age at time of the pneumococcal vaccination (SD)		84.0 (8.4)	
Median age at time of the pneumococcal vaccination (range)		84 (70–114)	

Age, years	70’s	1,217	35.6%
	80’s	1,247	36.4%
	90’s and over	958	28.0%

Sex	Male	1,483	43.3%
	Female	1,939	56.7%

Body mass index, kg/m^2^	<18.5	278	8.1%
	18.5–25	2,400	70.1%
	≧25	727	21.2%
	Unknown	17	0.5%

Smoking history	Current smoker	218	6.4%
	Past smoker	848	24.8%
	Never smoker	2,333	68.2%
	Unknown	23	0.7%

Smoking history of family members	Yes	625	18.3%
	No	2,754	80.5%
	Unknown	43	1.3%

Past history of pneumococcal vaccination	Yes	90	2.6%
	No	3,320	97.0%
	Unknown	12	0.4%

Past history of influenza vaccination	Yes	2,888	84.4%
	No	512	15.0%
	Unknown	22	0.6%

Respiratory diseases	Yes	298	8.7%
	-Asthma	124	3.6%
	-Chronic obstructive pulmonary disease	72	2.1%
	No	3,113	91.0%
	Unknown	11	0.3%

Cardiovascular diseases	Yes	401	11.7%
	-Angina pectoris	144	4.2%
	-Myocardial infarction	55	1.6%
	No	3,010	88.0%
	Unknown	11	0.3%

Other comorbidities	Hypertension	1,561	45.6%
	Hyperlipidemia	551	16.1%
	Diabetes mellitus	449	13.1%
	Stroke	147	4.3%

Nursing home admission	Yes	38	1.1%
	No	3,376	98.7%
	Unknown	8	0.2%

Performance status	0: Fully active, no restrictions	2,886	84.3%
	1: Limited in strenuous activity, able to do light work	357	10.4%
	2: Ambulatory, self-care, no work	94	2.7%
	3: Limited self-care, in bed/chair >50% of the time	43	1.3%
	4: Completely disabled, bedridden or chair-bound	23	0.7%
	Unknown	19	0.6%

SD, standard deviation.

### Incidence of pneumonia in individuals who participated in the pneumococcal vaccination support project

Among the 3,422 subjects who received the pneumococcal vaccine, 295 developed pneumonia during the 5-year observation period, which lasted until March 2020 at the latest. Participation in the annual follow-up surveys was excellent, with 99% of participants completing at least one survey, 73% followed for the full 5 years, and a total of 14,559 person-years of follow-up. Yearly follow-up loss rates, as shown in eTable 2, were 1.3% in the first year, 3.6% in the second, 4.7% in the third, 5.8% in the fourth, and 12.1% in the fifth year.

**Figure 3.**
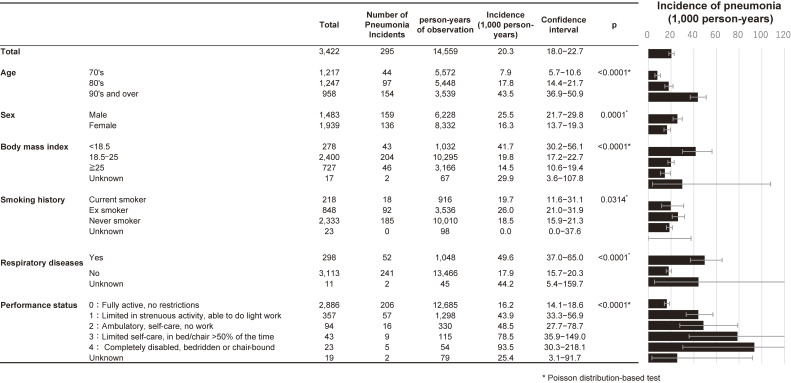
Incidence of pneumonia (1,000 person-years) in 3,422 residents aged 65 years and older in Sera Town who received the pneumococcal vaccine

Pneumonia incidence post-vaccination was 20.3/1,000 person-years (95% CI, 18.0–22.7/1,000 person-years) (Figure 3). Incidence rates were significantly higher in older age groups: 7.9/1,000 person-years (95% CI, 5.7–10.6/1,000) in those in their 70s, 17.8 (95% CI, 14.4–21.7/1,000) in their 80s, and 43.5 (95% CI, 36.9–50.9/1,000) in those aged 90 years and older. Males had higher incidence rates than females (25.5 vs 16.3/1,000 person-years). By BMI, the incidence of pneumonia was significantly higher in those with lower BMI. By smoking history, past smokers had the highest incidence of pneumonia at 26.0/1,000 person-years. By respiratory disease status, the incidence of pneumonia was 49.6/1,000 person-years in those with respiratory disease, which was significantly higher than that in those without respiratory disease (17.9/1,000 person-years). The incidence of pneumonia significantly varied by PS. Among those with no activity limitations (PS = 0), the rate was 16.2/1,000 person-years, whereas it was 55.5/1,000 person-years for individuals with PS 2 or higher. For individuals spending more than 50% of their day bedridden (PS = 3), the rate was 78.5/1,000 person-years, and for those completely bedridden (PS = 4), it was 93.5/1,000 person-years.

### Comparison of crude mortality rates in Sera Town with Hiroshima Prefecture and nationwide Japan

**Figure 4.**
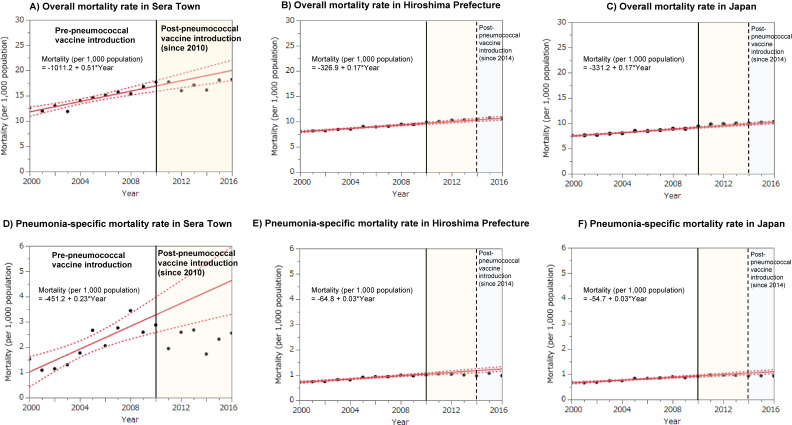
The discrepancy between crude mortality rates and predicted mortality rates based on data from 2000 to 2009 (pre-pneumococcal vaccine introduction in Sera Town). Circles indicate observed crude mortality rates, while red lines represent predicted mortality rates based on measured crude mortality rates from 2000 to 2009 (linear regression).

Based on the distribution of overall and pneumonia-specific crude mortality rates for the 10 years (2000–2009) before the introduction of the pneumococcal vaccination support project in Sera Town, a linear regression model was used to predict the mortality rate had the project not been introduced, and the results were compared with the actual mortality rates after 2010. The results are shown in Figure 4. The mortality rates for Hiroshima Prefecture and Japan were similarly predicted using a linear regression model based on the distribution of mortality rates from 2000 to 2009 and compared with the actual mortality rates from 2010 to 2016. Compared to Hiroshima Prefecture and nationwide Japan, Sera Town has a higher aging rate (40.3% in Sera Town, 27.5% in Hiroshima Prefecture, and 26.7% in Japan as of 2015). From 2000 to 2009, the overall crude mortality rate and pneumonia-specific crude mortality rate in Sera Town increased more rapidly than in Hiroshima Prefecture and nationwide Japan. However, since the project’s inception in 2010, both the overall crude mortality rate and the pneumonia-specific mortality rate in Sera Town have decreased and are significantly lower than predicted levels.

### Association of the pneumococcal vaccination support project with pneumonia-specific mortality in Sera Town using interrupted time series analysis

**Figure 5.**
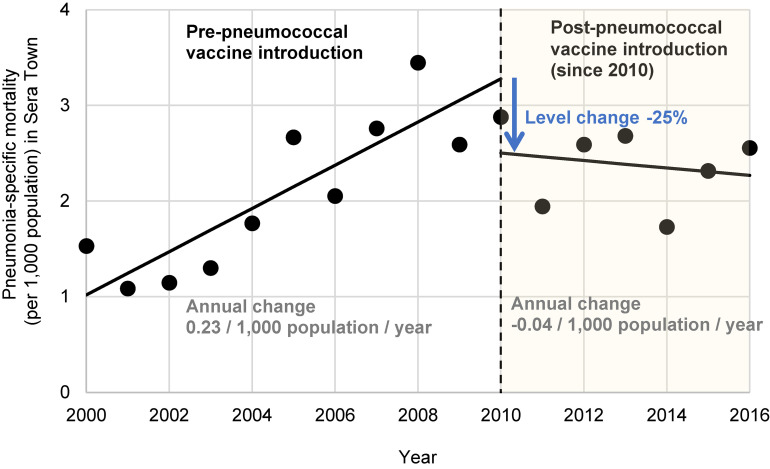
Change in the level and trend of pneumonia-specific mortality (per 1,000 population) in Sera Town from 2000 to 2016

The results of the ITSA are shown in Figure 5 and eTable 3. The pneumococcal vaccination support project reduced the pneumonia-specific mortality rate in Sera Town by 25%. Before the start of the project, the mortality rate of pneumonia increased at a rate of 0.23/1,000 population per year, but after the start of the project, the rate turned to a decreasing trend at a rate of 0.04/1,000 population per year.

## DISCUSSION

This community-based study evaluated the association between the introduction of pneumococcal vaccination, PPSV23, for elderly residents in Sera Town and the incidence and mortality rates of pneumonia. Sera Town is located in a rural area of Hiroshima Prefecture, with a population of approximately 17,000 and an aging rate exceeding 40%. The incidence of pneumonia following pneumococcal vaccination was analyzed using individual data from vaccine recipients over a 5-year follow-up period. The findings revealed that the incidence rate of pneumonia in the elderly population (median age: 84 years, range: 70–114 years) post-vaccination was 20.3 per 1,000 person-years (95% CI, 18.0–22.7 per 1,000 person-years). Regarding the efficacy of the pneumococcal vaccine (PPSV23), a placebo-controlled randomized, double-blind study of elderly care facility residents in Japan reported a pneumonia incidence rate of 55 per 1,000 person-years in the vaccinated group, compared to 91 per 1,000 person-years in the placebo group.^9^ In our study, when limited to subjects with a PS of 2 or higher, the pneumonia incidence rate was 55.5 per 1,000 person-years, approximating the results of the aforementioned randomized controlled trial (RCT). This study is unique in that it was a community-based study conducted in a specific town, and real-world data were analyzed for 84.3% of the total elderly population with no disability (PS = 0) in their daily lives. The observation period for this study extended up to March 2020, when the coronavirus disease 2019 (COVID-19) outbreak in Japan was still limited. At that time, only 6 cumulative cases had been reported in Hiroshima Prefecture, making the impact of COVID-19 pneumonia negligible. The results of the stratified analysis also revealed the incidence of pneumonia after pneumococcal vaccination by age, sex, BMI, presence of respiratory disease, and PS. These epidemiological findings are expected to be useful as basic data when considering the introduction of a pneumococcal vaccination program for the elderly general population. However, the type of pneumococcal vaccine must also be considered. This study focuses on PPSV23, which is currently used in Japan’s National Immunization Program. In 2011, PPSV23 serotypes accounted for 71.4% of cases in Japan, indicating that these serotypes were predominant at the beginning of this study.^10^ Since then, however, the serotype distribution has shifted, with the proportion decreasing to 50.0% by 2015. When evaluating PPSV23 effectiveness, regional serotype prevalence must be considered. Recent reports recommend PCV15 or PCV20, which are considered more effective, for first-time recipients aged 65 years and older.^11^ Furthermore, with the introduction of PCV13 in children, some reports suggest that due to serotype replacement and herd immunity, PPSV23’s effectiveness in the elderly may be limited.^11^^–^^13^ In Japan, the national expert committee managed by the Ministry of Health, Labour and Welfare is currently discussing whether to include pneumococcal vaccines, such as PCV20, for the elderly in the National Immunization Program.^14^ The pneumonia incidence following PCV15 or PCV20 vaccination in the elderly may be lower than that observed in this study.

Next, regarding mortality rates, while individual-level data analysis was not feasible, we conducted an analysis based on vital statistics open data (aggregated data). By comparing predicted values from a linear model with observed values and performing ITSA, we elucidated the mortality reduction effect of the pneumococcal vaccination support program on the entire community population. Before the project began, Sera Town, characterized by a high aging population, exhibited higher overall and pneumonia-specific crude mortality rates compared to those of Hiroshima Prefecture and the national average in Japan. Additionally, these rates were increasing annually. However, following the project’s commencement, there was a notable 25% decrease in pneumonia-specific mortality rates, marking a shift in the mortality trend from an increasing to a decreasing trajectory. Aside from the pneumococcal vaccination program for the elderly, other factors that may have contributed to the decline in mortality include the promotion of basic infection prevention behaviors through proactive pneumococcal vaccine campaigns in Sera Town, potential recommendations for influenza vaccination by physicians during pneumococcal vaccination, and the indirect effects of the nationwide introduction of PCV13 for children in 2013.^15^^–^^17^ ITSA is a method of analysis that allows estimation of intervention effects without the influence of individual-level confounding and can be considered a RCT with time as the allocation variable.^5^^–^^8^ It evaluates the overall impact of multiple co-occurring factors. In this context, while other factors may have had some influence, it is most reasonable to conclude that the pneumococcal vaccination program had the most significant impact on reducing mortality.

In Japan, which currently has the world’s most aged population, addressing pneumonia among the elderly is an exceptionally critical challenge. Elderly individuals face an elevated risk of pneumonia due to factors such as compromised immune function, physiological changes in the respiratory system, and the presence of comorbidities, all of which increase susceptibility to severe illness.^18^^,^^19^ Additionally, pneumonia can lead to declines in cognitive function and activities of daily living,^20^^,^^21^ thereby increasing the burden of caregiving on families. Associated healthcare costs are substantial,^22^ exerting a significant impact on society as a whole. Pneumococcal vaccination for the elderly has been reported to prevent pneumonia, reduce the severity of illness, prevent mortality, and control healthcare costs.^23^^–^^26^ As a result, many developed countries currently recommend pneumococcal vaccination for the elderly.^27^ In Japan, a pneumococcal vaccination program for individuals aged 65 years and older was introduced nationwide in 2014. However, the vaccination coverage remains at around 20%.^4^ This study provides real-world evidence of the mortality reduction achieved by administering pneumococcal vaccines to approximately 40% of the elderly population in Sera Town, a super-aged region with an aging rate exceeding 40%. In recent years, rapid aging has been occurring globally, particularly in Asian countries, making pneumonia prevention in the elderly a critical global issue. The practices demonstrated in Sera Town can provide valuable evidence, especially for regions where PPSV23-covered serotypes are prevalent, supporting other countries and areas considering pneumococcal vaccination programs for the elderly.

This study has several limitations. Regarding pneumonia incidence rates, the data are derived from a self-administered mail survey, which necessitates consideration of voluntary response bias and recall bias. Additionally, the absence of individual mortality data suggests the possibility that some participants lost to follow-up may have died from pneumonia, which was not accounted for. Nevertheless, the survey’s exceptionally high response rate, as previously noted, indicates that the impact of such biases is likely minimal. Sera Town, characterized by its strong community bonds, witnessed a remarkably high cooperation rate in this study, largely due to the established trust between the town officials leading the survey and the participating residents. Furthermore, since pneumonia is a significant health event for individuals, the influence of recall bias is expected to be also minimal. However, a limitation persists due to the absence of corroborating test results from medical institutions, which prevents confirmation of whether reported cases of “pneumonia” were specifically caused by Streptococcus pneumoniae. Regarding mortality, the study’s limitation is the lack of individual-level data on pneumonia-related deaths. However, leveraging aggregated open data allowed us to visualize the effect of mortality reduction.

In conclusion, this community-based study conducted in an extremely aging town provides real-world evidence on the incidence and mortality rates associated with pneumococcal vaccination. These findings are valuable for the aging global population and warrant sharing.

## SUPPLEMENTARY MATERIAL

eTable 1. Mean age of participants by year of pneumococcal vaccination

eTable 2. Participant numbers and follow-up loss rates over 5 years

eTable 3. Results of multivariate linear regression model for interrupted time series for pneumonia-specific mortality in Sera Town
