# Erratum for “Association Between Introduction of the 23-valent Pneumococcal Polysaccharide Vaccine (PPSV23) and Pneumonia Incidence and Mortality Among General Older Population in Japan: A Community-based Study”

**DOI:** 10.2188/jea.JE20250341

**Published:** 2026-02-05

**Authors:** Aya Sugiyama, Masaaki Kataoka, Kentaro Tokumo, Kanon Abe, Hirohito Imada, Bunlorn Sun, Golda Ataa Akuffo, Tomoyuki Akita, Shingo Fukuma, Noboru Hattori, Junko Tanaka

**Affiliations:** 1 Department of Epidemiology, Infectious Disease Control and Prevention, Graduate School of Biomedical and Health Sciences, Hiroshima University, Hiroshima, Japan; 2 Project Research Center For Epidemiological & Mega-Data Analysis of New Research Area, Hiroshima University, Hiroshima, Japan; 3 Sera Central Public Hospital, Hiroshima, Japan; 4 Department of Molecular and Internal Medicine, Graduate School of Biomedical and Health Sciences, Hiroshima University, Hiroshima, Japan; 5 Hiroshima University Hospital, Department of Clinical Oncology, Hiroshima, Japan

We would like to make a minor correction to our published article^1^ to clarify the interpretation of pneumococcal vaccination coverage cited in Reference 4.^2^ In the Introduction (page 237) and Discussion (page 243), we previously stated that the pneumococcal vaccination coverage rate was “between 19.8% and 21.7%.” However, these figures reflect the annual vaccination rates among newly eligible individuals (ie, those turning 65, 70, 75, 80, 85, 90, and 95 years) during the period from 2019 to 2021. Upon further consideration, we recognize that the cumulative coverage as of the end of fiscal year 2021, also reported in Reference 4, is more appropriate for evaluating the overall spread of vaccination in the elderly population. Therefore, we request that the following revisions be made:

Corrections

Page 237 (Introduction)
Original:
“However, the vaccination coverage rate is reported to be between 19.8% and 21.7%.^4^”
Revised:
“The vaccination coverage rate was reported to be between 19.8% and 21.7% during 2019–2021 among newly eligible individuals, although the cumulative coverage as of the end of fiscal year 2021 was estimated at 33.6–49.1%.^4^”

Page 243 (Discussion)
Original:
“However, the vaccination coverage remains at around 20%.^4^”
Revised:
“The vaccination coverage was reported to be around 20% among newly eligible individuals during 2019–2021, although the cumulative coverage as of the end of fiscal year 2021 was estimated at 33.6–49.1%.^4^”

We apologize for any confusion caused by the previous phrasing and appreciate the opportunity to correct the published record.
