# Supplementary material for: Association Between Introduction of the 23-valent Pneumococcal Polysaccharide Vaccine (PPSV23) and Pneumonia Incidence and Mortality Among General Older Population in Japan: A Community-based Study
Source: J Epidemiol. 2025 May 5;35(5):237–44. doi: 10.2188/jea.JE20240285 (PMC11979344; doi:10.2188/jea.JE20240285)
Supplement: Supplementary file 1 [file je-35-237-s001.pdf]

eTable 1. Mean age of participants by year of pneumococcal vaccination

Year of pneumococcal vaccination	Mean age, years	Standard deviation
2010	89.5	6.6
2011	88.1	6.9
2012	85.5	7.8
2013	80.9	7.3
2014	77.6	6.9
2015	78.9	7.5
Total	84.0	8.4

eTable 2. Participant numbers and follow-up loss rates over 5 years

	Number of participants followed up	Number of participants lost to follow-up	The follow-up loss rates
Follow-up year 0	3,422		
Follow-up year 1	3,379	43	1.3%
Follow-up year 2	3,257	122	3.6%
Follow-up year 3	3,095	162	4.7%
Follow-up year 4	2,898	197	5.8%
Follow-up year 5	2,483	415	12.1%
Total			27.4%

eTable 3. Results of multivariate linear regression model for interrupted time series for pneumonia-specific mortality in Sera Town

	Parameter	Estimate	SE	95% CI		P value
β_0	Intercept	1.02	0.26	0.45	1.58	0.0018
β_1	Y	0.23	0.05	0.12	0.33	0.0005
β_2	Z	1.87	1.13	-0.57	4.32	0.1222
β_3	YxZ	-0.27	0.10	-0.47	-0.06	0.0172

CI, confidencial interval; SE, standard error.

Y is year

Z is defined as a function that equals 1 when post-project and is otherwise equal to 0

Y×Z is an interaction term between year and Z. Thus β_3 provides a measure of the difference in the trend in mortality rate before and after the launch of the project.
